# Preparation of a functional fluorescent human Fas ligand extracellular domain derivative using a three-dimensional structure guided site-specific fluorochrome conjugation

**DOI:** 10.1186/s40064-016-2673-8

**Published:** 2016-07-07

**Authors:** Michiro Muraki

**Affiliations:** Biomedical Research Institute, National Institute of Advanced Industrial Science and Technology (AIST), Central 6, 1-1-1 Higashi, Tsukuba, 305-8566 Japan

**Keywords:** Human Fas ligand, Extracellular domain, Fluorochrome, Site-specific conjugation, Three-dimensional structure, Receptor-binding activity

## Abstract

**Background:**

Human Fas ligand extracellular domain has been investigated as an important target protein in the field of medical biotechnology. In a recent study, the author developed an effective method to produce biologically active human Fas ligand extracellular domain derivatives using site-specific chemical modifications.

**Findings:**

A human Fas ligand extracellular domain derivative containing a reactive cysteine residue within its N-terminal tag sequence, which locates not proximal to the binding interface between the ligand and the receptor in terms of the three-dimensional structure, was modified by Fluorescein-5-Maleimide without impairing the specific binding activity toward human Fas receptor extracellular domain. The purified protein sample free of low molecular-weight contaminants showed a characteristic fluorescence spectrum derived from the attached Fluorescein moieties, and formed a stable binding complex with human Fas receptor extracellular domain—human IgG_1_ Fc domain fusion protein in solution. The conjugation number of the fluorochrome was estimated to be 2.5 per a single human Fas ligand extracellular domain trimer from the ratio of the absorbance value at 280 nm to that at 495 nm.

**Conclusions:**

A functional fluorescent human Fas ligand extracellular domain derivative was prepared via a site-specific conjugation of fluorochrome, which was guided by the three-dimensional structure information on the ligand-receptor complex. Fluorescent derivatives created by this method may contribute to the development of an improved diagnosis system for the diseases related to Fas receptor.

**Electronic supplementary material:**

The online version of this article (doi:10.1186/s40064-016-2673-8) contains supplementary material, which is available to authorized users.

## Background

Fas ligand-induced apoptosis plays an essential role in maintaining human health, and loss of function of this system directly leads to the onset of serious diseases (Nagata 1999). Therefore, this protein has been considered as an important target for the therapy of diseases, especially cancers and immunologic disorders (Linkermann et al. 2005; Villa-Morales and Fernández-Piqueras 2012). Accordingly, a number of engineered proteins concerning human Fas ligand (CD178) were devised for possible treatment of such diseases to date (Villa-Morales and Fernández-Piqueras 2012; Wajant et al. 2013). Also, the clinical significance of soluble form of the counterpart receptor, human Fas receptor (CD95), as a potentially useful diagnostic biomarker has been repeatedly pointed out in medical evaluation studies on a variety of cancers (Holdenrieder and Stieber 2004). The overall potential of human Fas ligand extracellular domain (hFasLECD) for various applications in the field of medical biotechnology concerning therapeutic relevance, sample preparation and structure–function relationships was summarized in a recent review paper (Muraki 2014a).

Chemical modification is a powerful methodology for creating artificially engineered proteins with novel, useful properties, which are not available in the original protein molecules (Chalker et al. 2011). On the other hand, conducting the chemical modifications raises the possibility of impairment of valuable biological activity of the original proteins, if randomly performed. In a recent study, the author developed a new method to introduce chemical modifications into an hFasLECD derivative via the conjugation of a reactive cysteine residue existing in the N-terminal tag sequence with a maleimide group containing compounds (Muraki 2014b). The modifications were able to be carried out without damaging the original biological functions including apoptosis inducing activity of hFasLECD in the presence of a cross-linking antibody.

In the present study, the conjugation with a maleimide group containing derivative of one of the most popular fluorochromes in cell-biological researches, Fluorescein-5-Maleimide (FL-5Mal), was examined as an extension of the above mentioned method to explore the possibility of preparing functional fluorescent derivatives of hFasLECD.

## Methods

### Materials

hFasLECD containing single deletion mutation from 103 to 138 and double substitution mutations (N184Q and N250Q) [hFasLECD (139-281, N184Q, N250Q)] with an N-terminal FLAG-(GlyCysGlyGlyGlyGly) tag sequence (NFG1CG4-hFasLECD) and that with an N-terminal FLAG-(Gly)_5_ tag sequence (NFG5-hFasLECD) were prepared as described (Muraki 2008, 2014b). FL-5Mal was obtained from Tokyo Chemical Ind. Dimethyl Sulfoxide, Super Dehydrated (Dry DMSO), l-Cysteine hydrochloride monohydrate and pre-cast gels for SDS-PAGE analysis (Supersep Ace, 10–20 % gradient gels) were from Wako Pure Chemicals Ind. BCA protein assay kit and Tris-(2-carboxyethyl)phosphine (TCEP) neutral pH solution were purchased from Thermo Fisher Scientific. Ion-exchange chromatography and size-exclusion chromatography were performed using prepacked columns from GE healthcare. Ultracentrifugation devices for sample concentration [Amicon Ultra 4 and 15, molecular-weight cut off (MWCO): 10 kDa] were supplied from Merck Millipore. Phosphate, acetate and Tris-hydrochloride buffers were the products of Nakarai Tesque (the former two) and Nippon Gene, respectively. SureBeads Protein A Magnetic Beads and washing buffer reagents used in immunoprecipitation experiments were from Bio-Rad Laboratories and Roche Diagnostics. Chemical structure of FL-5Mal was drawn using ChemBioDraw Ultra, ver. 14.

### Reaction of NFG1CG4-hFasLECD with FL-5Mal

The recombinant NFG1CG4-hFasLECD was produced by a *Pichia pastoris* secretion system. The sample used for the modification with FL-5Mal was pre-purified by cation-exchange chromatography (Hi-Trap SP HP) preceding the conjugation reaction as described (Muraki 2014b), and protein concentration of the purified sample was determined to be 7.6 mg/ml by a BCA protein assay kit using bovine serum albumin as a standard. The solution (1.5 ml) containing 11.4 mg protein was first mixed with 30 μl of 0.5 M Ethylenediaminetetracetic acid sodium salt (EDTA Na) solution (pH 8.0) and then treated with 30 μl of 0.5 M TCEP solution (neutral pH), which gave a final reaction mixture containing 10 mM each of EDTA Na and TCEP. The reaction mixture was incubated for 1 h at 297 K and subsequently subjected to a PD-10 size-exclusion chromatography column, which was pre-equilibrated and resolved using 25 mM phosphate buffer plus 2 mM EDTA Na (pH 6.4) as the eluent. The elution fraction (3.5 ml) containing the reduced NFG1CG4-hFasLECD after removing the excess amount of TCEP was diluted with 6.2 ml of the same buffer. Then, freshly prepared 20 mM FL-5Mal solution in Dry DMSO (0.97 ml) was added, and incubated for 1 h at 297 K to give a fluorescent deep-orange solution. After 1 h, the excess FL-5Mal was quenched by adding 19 μl of 1 M l-Cysteine hydrochloride, which immediately turned the color of the reaction mixture to deep-yellow showing stronger fluorescence, and further incubated for 1 h at 297 K. During all the reaction time after the addition of FL-5Mal, a polypropylene vessel used in the reactions was wrapped with aluminum foil for shading from light as possible.

### Purification of FL-5Mal conjugated NFG1CG4-hFasLECD

Purification of NFG1CG4-hFasLECD conjugated with FL-5Mal was conducted by three steps of size-exclusion chromatography. In the former two steps, a PD-10 column and 50 mM sodium acetate buffer (pH 5.5) were used as the chromatography device and the elution buffer in a gravity flow mode, respectively. In the first resolving step, a 2.5 ml each aliquot of the quenched conjugation reaction mixture was applied to the column and one ml each fraction was collected into the reservoirs. The early five fractions starting from the second fraction eluted as clear yellow solutions, which showed weaker fluorescence than the seventh and the following fractions emitting much stronger lemon-yellow fluorescence. Only the four elution fractions (from the second to the fifth fraction) containing the conjugated protein were combined together (total volume 22 ml) and concentrated to approximately 4.0 ml using an Amicon Ultra 15 (MWCO: 10 kDa) device. Then, a 2.0 ml each aliquot was subjected to the second resolving step using a PD-10 column for the purpose of removing the low-molecular weight contaminants completely. At this stage, the yield of recovered protein was 5.3 mg in 7.0 ml solution. This sample was used for the final purification step by high-performance size-exclusion chromatography (used column: Superdex 200 10/300 GL, bed dimensions: 10 × 300 mm, bed volume: approximately 24 ml). In this step, 50 mM Tris-hydrochloride containing 150 mM sodium chloride (pH 7.5) was employed as the elution buffer. The flow rate was set to 0.5 ml/min and a 230 μl each aliquot of the sample was applied to the column for an individual run. Two significant peaks at identical positions were detected by the measurement of absorbance at both 280 and 495 nm, and each peak fractions were collected independently. After concentration using an Amicon Ultra 4 (or 15) centrifugation device (MWCO: 10 kDa), the collected samples were subjected to examine the content by a SDS-PAGE analysis. The same sample was also examined in the complex formation experiments with human Fas receptor extracellular domain—human IgG_1_ Fc domain (hFasRECD-Fc) using immunoprecipitaion and size-exclusion chromatography analyses.

### Production and purification of hFasRECD-Fc

The recombinant hFasRECD-Fc protein was produced in a baculovirus—*Bombyx mori* larvae expression system, and purified using an affinity chromatography column (Hi-Trap Protein G HP) and an anion-exchange chromatography column (Mono Q 10/10) as described in the previous paper (Muraki and Honda 2010).

### Spectroscopic measurements

Ultraviolet–visible (UV–Vis) absorbance spectra were recorded on a Bio-Rad SmartSpec Plus Spectrophotometer in the range from 250 to 600 nm using a two-sides clear quartz cuvette of 1 cm light-path length. A couple of absorbance values at 280 and 495 nm were also measured independently for the calculation of an estimated number of the conjugated FL-5Mal moieties to NFG1CG4-hFasLECD. Fluorescence spectra were recorded on a KONTRON SFM25 spectrofluorometer under the conditions of the excitation wavelength at 495 nm and the emission wavelength scan range from 700 to 400 nm using a four-sides clear quartz cuvette of 1 cm excitation light-path length. Protein samples of 0.125 mg/ml concentration in 50 mM Tris-hydrochloride buffer containing 150 mM sodium chloride (pH 7.5) were used for the measurements at 298 K.

### Estimation of the conjugation number

The number of FL-5Mal moieties conjugated to NFG1CG4-hFasLECD was estimated according to the calculation equations described in the instruction manual of Pierce FITC Antibody Labeling kit (Thermo Fisher Scientific Inc. 2014) from the measured absorbance values at 280 and 495 nm. Molar extinction coefficient of Fluorescein group at 495 nm and that of NFG1CG4-hFasLECD at 280 nm were assumed as 70,000 and 29,005, respectively. The latter value was obtained using the Prot Param tool on the ExPASy Server (Gasteiger et al. 2005).

### Detection of the complex formation

Detection of the specific binding activity of the FL-5Mal conjugated NFG1CG4-hFasLECD samples (5.0 μg each) toward the hFasRECD-Fc sample (8.0 μg) was conducted using a Protein A conjugated magnetic beads (1.0 mg) by a receptor-mediated co-immunoprecipitation in 50 mM Tris-hydrochloride buffer (pH 7.5) containing 150 mM sodium chloride, 1 % Nonidet P40 and 0.5 % sodium deoxycholate (1.0 ml). The bound FL-5Mal conjugated NFG1CG4-FasLECD—hFasRECD-Fc complex on the beads was rigorously washed twice using the same buffer (1.0 ml). After the final washing with 10 mM Tris-hydrochloride buffer (pH 7.5) containing 0.1 % Nonidet P40 and 0.05 % sodium deoxycholate (1.0 ml), the bound protein complex was solubilized using a non-reducing SDS-PAGE sample loading buffer (40 μl) composed of 125 mM Tris-hydrochloride (pH 6.8), 4.3 % SDS, 30 % Glycerol and 0.01 % Bromophenol Blue dye by incubating at 343 K for 12 min.

Another experiment for the detection of complex formation between FL-5Mal conjugated NFG1CG4-hFasLECD and hFasRECD-Fc was conducted by high-performance size-exclusion chromatography. An initial attempt was performed by injecting a mixture of the Mono Q column purified hFasRECD-Fc sample (30 μg) and the PD-10 column purified FL-5Mal conjugated NFG1CG4-hFasLECD sample (22 μg). With regard to the more detailed examination using the main peak samples purified by a Superdex 200 column, the mixture solutions composed of hFasRECD-Fc (19.4 μg) and FL-5Mal conjugated NFG1CG4-hFasLECD (5.0, 7.5, 15 or 30 μg) in 230 μl solution were analyzed. In all cases, the resolution by a Superdex 200 10/300 GL size-exclusion chromatography column using 50 mM Tris-hydrochloride containing 150 mM sodium chloride (pH 7.5) as the elution buffer under the flow rate of 0.5 ml/min was employed for the analyses.

## Results and discussion

### Conjugation design

Recognition of death receptors by death ligands including Fas ligand has been considered as important targets for therapy of many serious human diseases such as cancers (Ashkenazi 2008; Russo et al. 2010), and the specific interaction between the extracellular domains of them is a fundamental to the recognition. The extracellular domains of the proteins belonging to death ligands and death receptors, also called as tumor necrosis factor (TNF) ligand superfamily and TNF receptor superfamily, share each common structural features in three-dimensions (Bodmer et al. 2002). To date, several detailed three-dimensional structures of the extracellular domain complexes, including human TNF receptor–human TNFβ complex (Banner et al. 1993) and human death receptor 5–human TNF related apoptosis inducing ligand complex (Hymowitz et al. 1999), have been elucidated by mean of crystallography. A typical structure of the extracellular domain complexes is composed of three monomers of the receptor’s extracellular domains and one trimer of the ligand’s extracellular domains. Although no three-dimensional structure of the extracellular domains complex between human Fas ligand and human Fas receptor itself has been experimentally disclosed yet, a detailed three-dimensional structure of the extracellular domains complex between human Fas ligand and human decoy receptor 3 (hDcR3), which was revealed by X-ray crystallography, is already available (Liu et al. 2013). This X-ray structural model provided important information for the design of the conjugation site in this study. Although the primary sequence identity between hFasR and hDcR3 is moderate (17 %, Pitti et al. 1998), the three-dimensional interaction modes with hFasLECD, forming the binding interfaces of the ligand-receptor complexes, are considered essentially conserved to each other (Whalen and Hymowitz 2014).

**Fig. 1:**
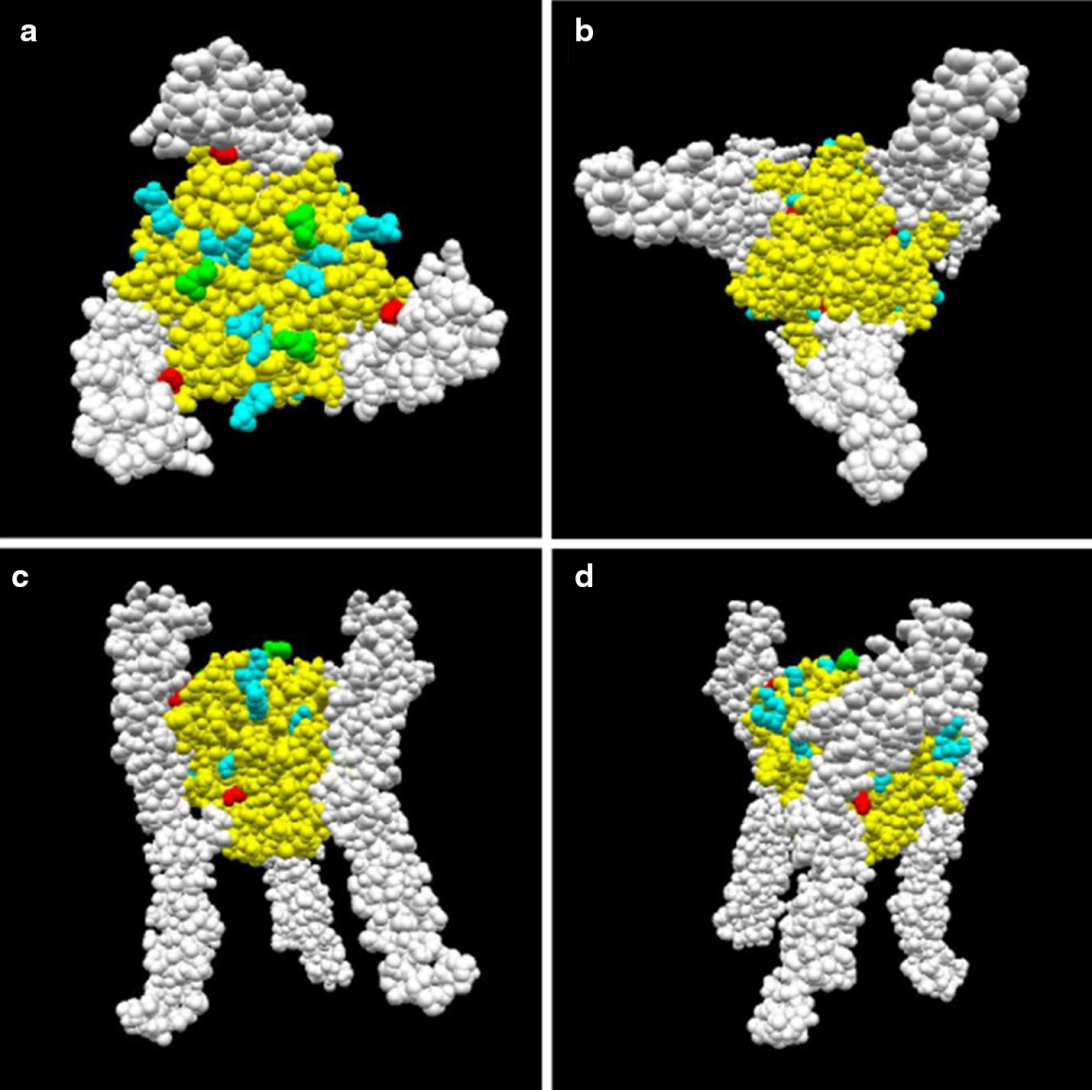
Three-dimensional structure of hFasLECD–hDcR3 complex. The atomic coordinate data were obtained from PDB (ID: 4msv). A biological unit image composed of a single hFasLECD trimer (aa 143–281, *yellow*) and a triply bound hDcR3 monomers (aa 33–193, *white*) is presented as a space-filling model. The N-terminal residue in this model (Leu 143), the two lysine residues mentioned in the text (Lys 218 and Lys 228) and the other lysine residues are shown in *green*, *red* and *cyan*, respectively. **a** A* vertical view*, **b** a* vertical view* (after 180 degree rotation of **a**), **c** a* horizontal view*, **d** a* horizontal view* (after 180 degree rotation of **c**)

Figure 1 presents the three-dimensional structure of the hFasLECD–hDcR3 complex (Liu et al. 2013). It was aimed to create a functional hFasLECD derivative containing Fluorescein residue in this study. Probably, the most popular strategy for introducing Fluorescein moieties into protein molecules, such as antibodies, is to modify the lysine residues present on the surface of the proteins by Fluorescein isothiocyanate (Thermo Fisher Scientific Inc. 2014). However, each NFG1CG4-hFasLECD monomer contains fourteen lysine residues including the two residues within the N-terminal FLAG tag sequence, and most of them locate on the molecular surface of the protein (Additional file 1). These lysine residues are often conserved among other animals’ Fas ligand extracellular domains (Motegi-Ishiyama et al. 2001). So far, two of them are suggested to be critical for the specific binding toward hFasRECD due to the existence of the residues at the binding interface. The side chain group of Lys 228 was predicted to form a salt-bridge with that of Asp 92 in hFasRECD from a molecular modeling study (Bajorath 1999). Lys 217 also locates in the contact site and is neighboring to Tyr 218, which was suggested to play an important role in the binding interactions from a mutagenesis study (Schneider et al. 1997). Therefore, a random modification of the lysine residues with fluorochrome moieties can significantly damage the receptor binding activity.

In contrast, only one unpaired cysteine residue in the N-terminal tag region and a single pair of disulfide-bridged cysteine residues within the folded region exist in NFG1CG4-hFasLECD. Judging from the position of the N-terminal residue (Leu143) in the three-dimensional structure of the hFasLECD–hDcR3 complex (Fig. 1) as well as from the fairly hydrophilic property of the N-terminal FLAG tag plus aa 139-142 region (Asp–Tyr–Lys–Asp–Asp–Asp–Asp–Lys–Gly–Cys–Gly–Gly–Gly–Gly–Glu–Lys–Lys–Glu), the unpaired Cys was expected to locate not proximal to the binding interface, but exposed to the solvent in an aqueous solution of neutral pH. Although the pre-activation by a treatment with some reducing agents was necessary for an efficient conjugation because of the propensity to form a disulfide-bridge between two NFG1CG4-hFasLECD monomer subunits due to non-specific oxidation of the unpaired cysteine residue, it was demonstrated that selective activation of the conjugation site alone was possible by choosing the mild reducing condition using TCEP for the reaction in the previous study (Muraki 2014b). Moreover, the resulting conjugates with several kinds of single or double maleimide group(s) containing compounds maintained the receptor binding activity, and also one conjugate was proved to exhibit significant cytotoxic activity against a cancer cell line after cross-linking by an anti-FLAG tag antibody (Muraki 2014b). Consequently, FL-5Mal was chosen as the modification reagent for the conjugation in this study.

### Preparation and characterization of FL-5Mal conjugated NFG1CG4-hFasLECD

**Fig. 2:**
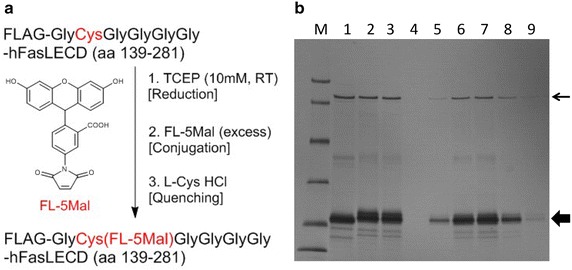
Conjugation of FL-5Mal to NFG1CG4-hFasLECD. **a** Experimental procedures. **b** SDS-PAGE analysis of the conjugation reaction and resolution by the first size-exclusion chromatography. *Lanes*: *M* molecular-weight size markers (14.4, 20.1, 30.0, 42.4, 66.3 and 97.4 kDa), *1* TCEP reduced sample before conjugation reaction, *2* after conjugation reaction, *3* after quenching reaction, *4* initial flow-through fraction (2.5 ml) in the first size-exclusion chromatography, *5*–*9* following fractions (1 ml each). Used column, PD-10. *Thick* and *thin arrows* indicate the positions of FL-5Mal conjugated hFasLECD and *P. pastoris* AOX-1, respectively

The conjugation procedures used in this study are summarized in Fig. 2a. In Fig. 2b, SDS-PAGE analysis of the reaction mixtures at each step (lanes 1–3) and the early six fractions in the first purification step using size-exclusion chromatography (lanes 4–9) are presented. The purified sample after a complete removal of low-molecular weight contaminants by the second size-exclusion chromatography still contained impurities shown as some minor bands less than 20.1 kDa and a sharp band between the size markers of 66.3 and 97.4 kDa arrowed in Fig. 2b. Judging from the band position, the latter component was considered to be the host-derived alcohol oxidase 1 (AOX-1) (molecular weight under the denaturing conditions: 75 kDa) (Zhang et al. 2009), which was co-purified from the culture medium used for the secretory production of NFG1CG4-hFasLECD in *P. pastoris* (Muraki 2014b).

**Fig. 3:**
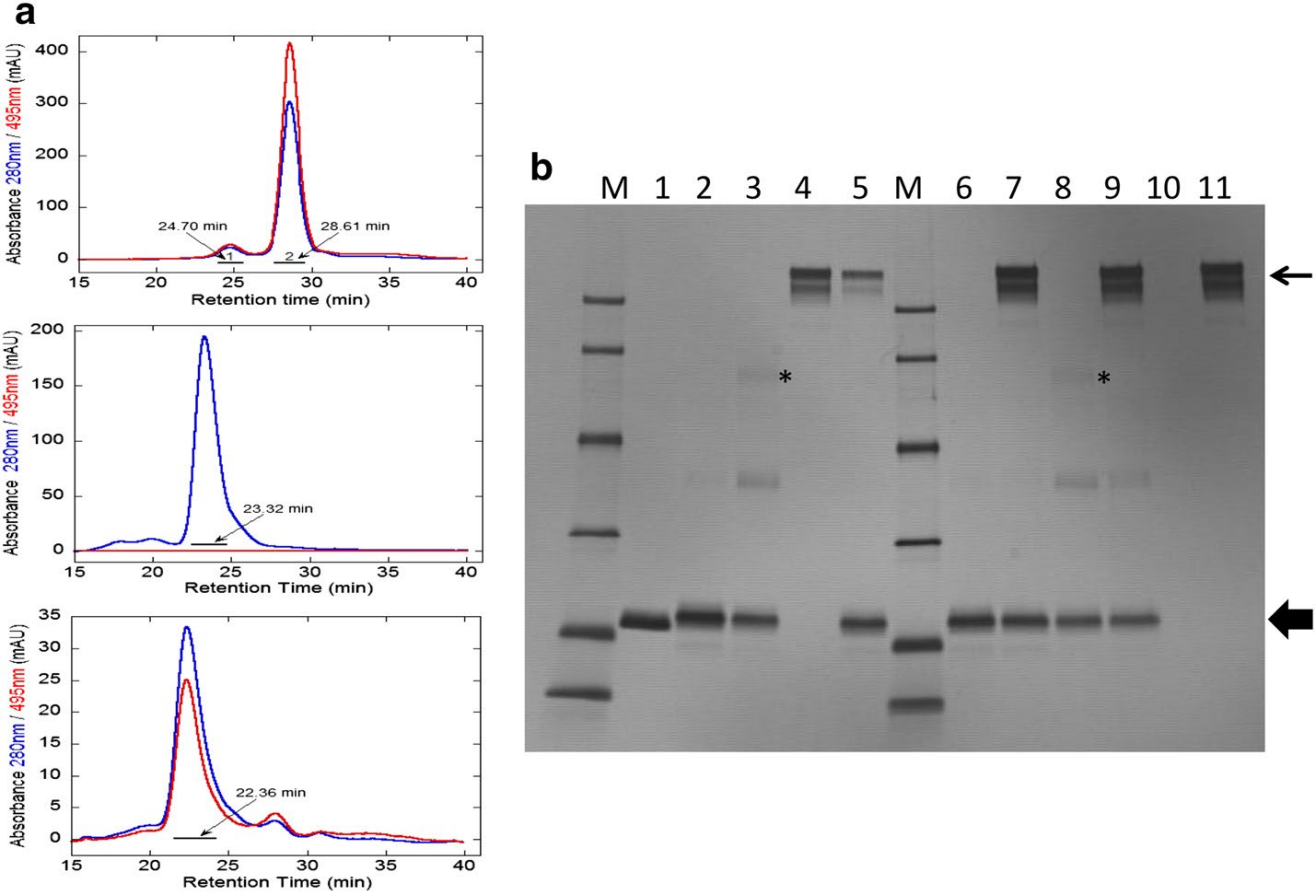
Purification using high-performance size-exclusion chromatography. **a** Elution profiles of the partially purified samples and a mixture of them. *Top panel* FL-5Mal conjugated NFG1CG4-hFasLECD; *Middle panel* hFasRECD-Fc; *Bottom panel* the mixture. Used column, Superdex 200 10/300 GL. Elution buffer, 50 mM Tris-hydrochloride plus 150 mM sodium chloride (pH 7.5). Absorbance at 280 nm (*blue*) and 495 nm (*red*) was used for the detection. Flow rate, 0.5 ml/min. The retention time of each peak position is shown. The regions shown in underbars were collected and used for the analysis in **b**. **b** SDS-PAGE analysis of the purified samples. *Lanes*: *M* molecular-weight size markers (14.4, 20.1, 30.0, 42.4, 66.3 and 97.4 kDa), *1*–*5* purified samples (1, NFG5-hFasLECD; *2* and *3* peak 2 and peak 1 of the FL-5Mal conjugated NFG1CG4-hFasLECD sample, respectively, *4* main peak of the hFasRECD-Fc sample, *5* main peak of the mixture sample), *6*–*11* receptor-mediated co-immunoprecipitation (*6* and *8* purified proteins, *10* buffer only, *7*, *9* and *11* co-immunoprecipitated materials; *6* and *7* peak 2 of the FL-5Mal conjugated NFG1CG4-hFasLECD sample, *8* and *9* peak 1 of the FL-5Mal conjugated NFG1CG4-hFasLECD sample, *10* and *11* the buffer only sample). *Thick* and *thin arrows* indicate the positions of FL-5Mal conjugated hFasLECD and hFasRECD-Fc, respectively

Figure 3a presents the high-performance size-exclusion chromatography profiles for the partially purified samples of FL-5Mal conjugated NFG1CG4-hFasLECD and hFasRECD-Fc, and a mixture of them. The conjugated NFG1CG4-hFasLECD sample after the second size-exclusion chromatography using a gravity flow column showed one major peak eluted at 28.61 min (peak 2) and a small preceding peak at 24.70 min (peak 1) (Fig. 3a, top panel) with respect to the absorbance at both 280 and 495 nm. The hFasRECD-Fc sample after the anion-exchange chromatography purification showed a main peak at 23.32 min presenting only the absorbance at 280 nm (Fig. 3a, middle panel). The mixture sample showed a main peak at an earlier position (22.36 min) than the receptor alone sample with regard to the absorbance at both 280 and 495 nm (Fig. 3a, bottom panel), indicating a stable complex formation derived from the strong interactions between them. The two independent peak fractions regarding the FL-5Mal conjugated NFG1CG4-hFasLECD sample, the main peak fraction concerning the hFasRECD-Fc sample and that of the mixture sample were collected and the components were analyzed by SDS-PAGE (Fig. 3b, lanes 2–5). In addition, the two peak fractions of the FL-5Mal conjugated NFG1CG4-hFasLECD sample were also examined by a receptor-mediated co-immunoprecipitaion experiment (Fig. 3b, lanes 6–9). The peak 2 sample showed an almost uniform molecular weight slightly larger than NFG5-hFasLECD (Fig. 3b, lane 1) due to the addition of FL-5Mal moiety and no longer contained the AOX-1 impurity (Fig. 3b, lanes 2 and 6), which was still observed in the sample after the second gravity flow size-exclusion chromatography purification. This sample precipitated with Protein A conjugated magnetic beads in the presence of hFasRECD-Fc, confirming the specific binding activity of the hFasLECD conjugate to the hFasRECD-Fc (Fig. 3b, lane 7). The peak 1 sample was a mixture of mainly three components, showing discrete molecular-weights (Fig. 3b, lanes 3 and 8). Interestingly, only the asterisked component showing the highest molecular-weight did not co-precipitated with the hFasRECD-Fc (Fig. 3b, lane 9). The main peak fraction obtained from the mixture sample contained both the ligand and the receptor components (Fig. 3b, lane 5). Under the same resolving conditions, molecular-weight standard samples of Thyrogloblin (669 kDa), Aldolase (158 kDa) and Ovalbumin (43 kDa) showed the peak retention time of 17.56, 24.28 and 29.20 min, respectively (Additional file 2). Purification of the FL-5Mal conjugated NFG1CG4-hFasLECD sample using a cation-exchange chromatography was not effective because of the broadening of the peak concomitant with a significant retardation probably due to the attached hydrophobic Fluorescein moieties (Additional file 3).

**Fig. 4:**
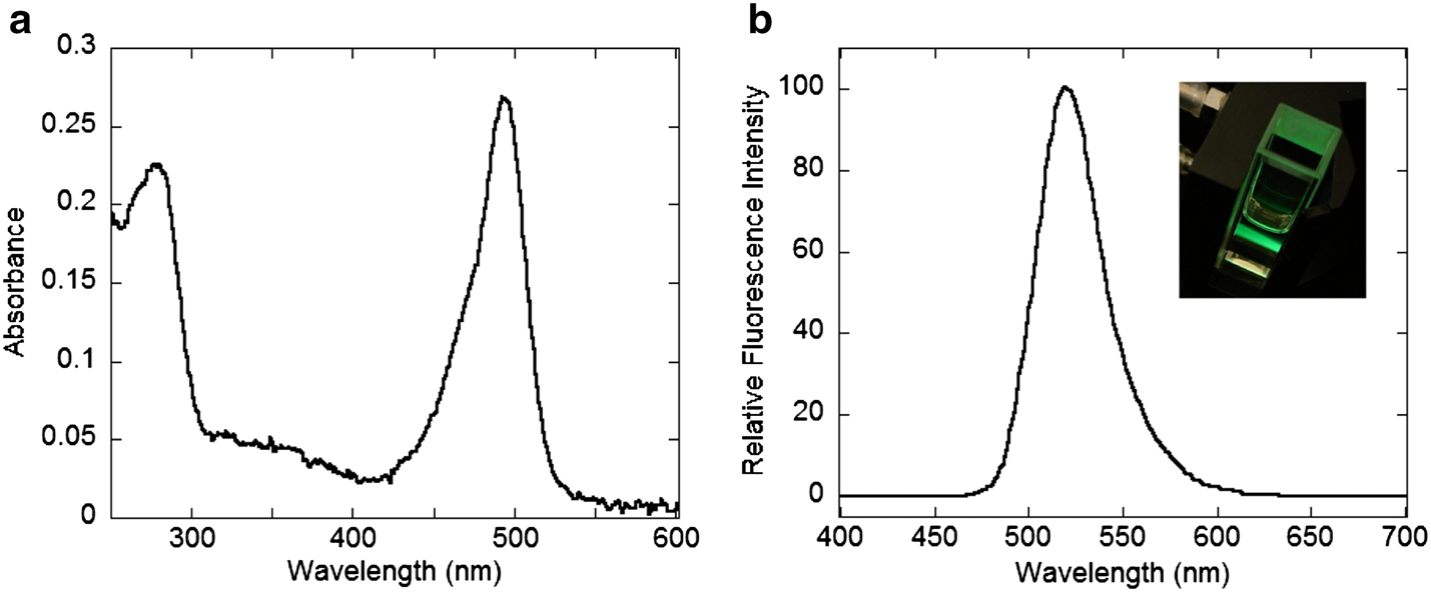
Spectroscopic analysis of FL-5Mal conjugated NFG1CG4-hFasLECD. The purified peak 2 sample of the FL-5Mal conjugated NFG1CG4-hFasLECD was used. **a** UV–Vis spectrum (250–600 nm). **b** Fluorescence emission spectrum (400–700 nm) excited at 495 nm. *Insert* a fluorescence emission observed in the measurement cuvette

The main peak sample of FL-5Mal conjugated NFG1CG4-hFasLECD purified by high-performance size-exclusion chromatography was then characterized using a couple of spectroscopic analyses. As shown in Fig. 4a, the sample presented two major peaks at 280 and 495 nm, which can be attributed to mainly from the protein part and the conjugated Fluorescein group, in the UV–Vis spectrum, respectively. From the ratio of the absorbance value at 280 nm to that at 495 nm, the conjugation number of FL-5Mal per a single NFG1CG4-hFasLECD trimer was estimated to be 2.5.

This result indicated that approximately 83 % of the unpaired cysteine residues in the NFG1CG4-hFasLECD sample were modified by the FL-5Mal molecules, since each single monomer constituting the trimer has one conjugation site within its N-terminal tag sequence. On the other hand, the measurement using a spectrofluorometer excited at 495 nm presented the characteristic emission spectrum derived from a Fluorescein moiety (BD Bioscience 2016) showing an intense yellow–green fluorescence with the maximum wavelength at 520 nm (Fig. 4b).

**Fig. 5:**
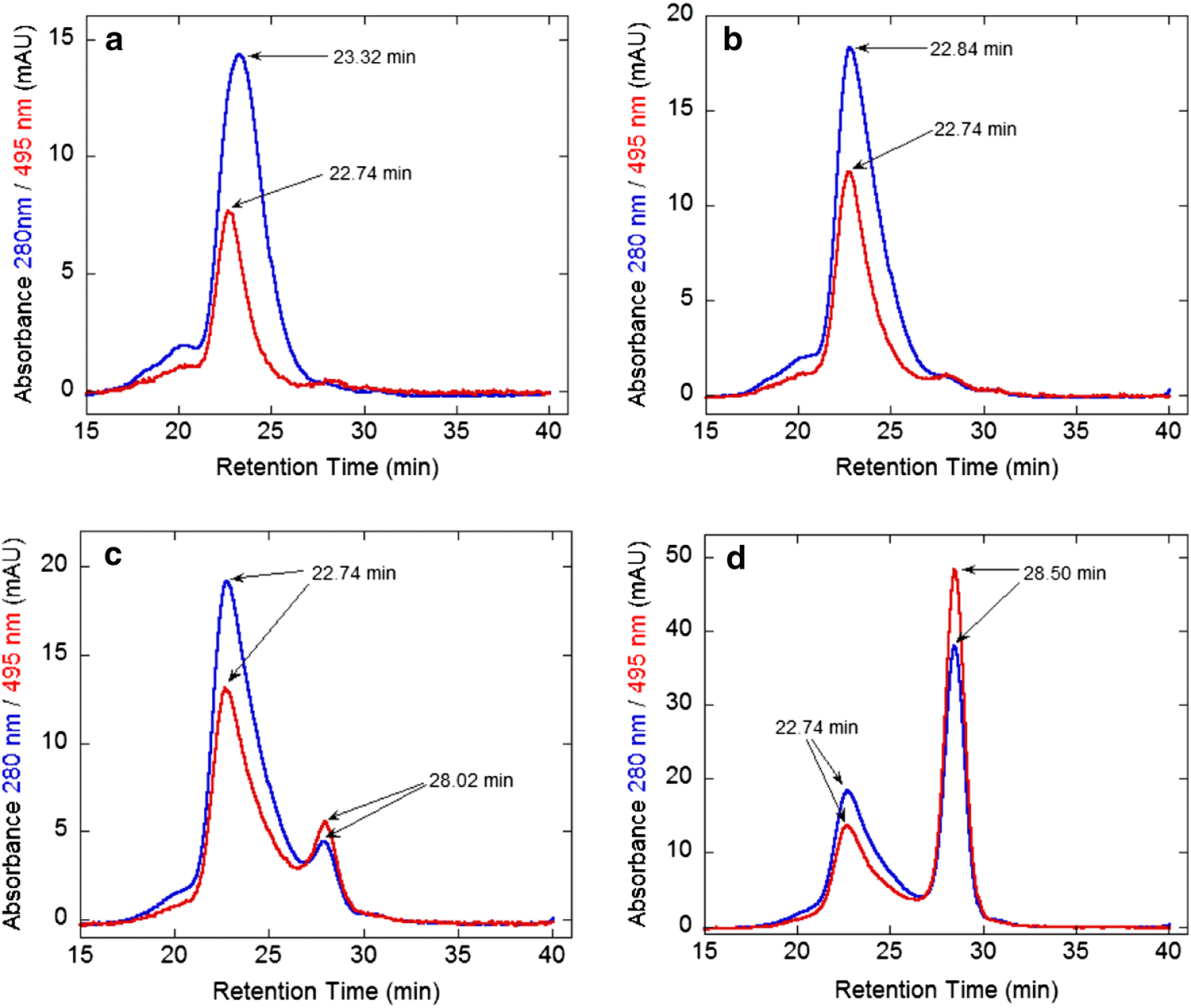
Analysis of the complex formation by high-performance size-exclusion chromatography. The mixtures of FL-5Mal conjugated NFG1CG4-hFasLECD (L) and hFasRECD-Fc (R) in four different amount ratios were examined. **a** L 5.0 μg and R 19.4 μg, **b** L 7.5 μg and R 19.4 μg, **c** L 15 μg and R 19.4 μg, **d** L 30 μg and R 19.4 μg. Used column, Superdex 200 10/300 GL. Elution buffer, 50 mM Tris-hydrochloride plus 150 mM sodium chloride (pH 7.5). Absorbance at 280 nm (*blue*) and 495 nm (*red*) was used for the detection. Flow rate, 0.5 ml/min. The retention time of each peak position is shown

Finally, the complex formation of FL-5Mal conjugated NFG1CG4-hFasLECD with hFasRECD-Fc was further examined more in detail by altering the relative mixing ratio of the main peak samples purified by the high-performance size-exclusion chromatography (Fig. 5, panels a–d). The 495 nm absorbance observed only with FL-5Mal conjugated NFG1CG4-hFasLECD was helpful in monitoring the elution position of this component. By gradually increasing the amount of FL-5Mal conjugated NFG1CG4-hFasLECD under the fixed amount of hFasRECD-Fc in the sample mixture, the 495 nm peak showing constant elution time (22.74 min) corresponding to the formed complex first became higher (panels a and b) and then the peak of the left over ligand component after saturation appeared at 28.02 min or 28.50 min (panels c and d). This phenomenon suggested that the peak fraction eluted at 22.74 min was consisted of the complex formed from a limited number of the ligand component and the receptor component.

## Conclusions and implications

In conclusion, it was possible to prepare a fluorescent derivative of hFasLECD using a site-specific chemical conjugation of FL-5Mal to NFG1CG4-hFasLECD without impairing the specific hFasRECD binding activity. This method will be readily applicable to other fluorochromes, if the corresponding maleimide derivatives are available. Fluorescent derivatives of ligand proteins and antibodies are useful for immunofluorescence imaging studies as well as flow cytometry analyses targeted to their counterpart receptors, especially those on the surface of viable cells (Schechter et al. 1978; Sako et al. 2000; Urano et al. 2009). It was suggested that many clinically relevant cytotoxic drugs such as cisplatin alter the susceptibility of a variety of tumor cells to apoptosis through the enhanced expression (Micheau et al. 1997; Galenkamp et al. 2015) and the clustering in lipid rafts (Lacour et al. 2004; Lim and Han 2015) of the cell-surface Fas receptor. The fluorescent derivative of functional hFasLECD created by the method described in this paper may contribute to the development of an improved diagnostic system as a new additional molecular tool accompanied by treatments of the disorders, in which the states of Fas receptor are reflected by the diseases’ conditions.

## Additional files

10.1186/s40064-016-2673-8 Stereo-view of three-dimensional hFasLECD structure.
10.1186/s40064-016-2673-8 Analysis of molecular-weight size markers by size-exclusion chromatography.
10.1186/s40064-016-2673-8 Cation-exchange chromatography profile of the purified sample.

## Abbreviations

hFasLECD: human Fas ligand extracellular domain
hFasR: human Fas receptor
hFasRECD-Fc: a fusion protein composed of human Fas receptor extracellular domain and human IgG_1_-Fc domain
hDcR3: human decoy receptor 3
TNF: tumor necrosis factor
aa: amino acid residues
FL-5Mal: Fluorescein-5-Maleimide
TCEP: tris-(2-carboxyethyl)phosphine
EDTA Na: ethylenediaminetetracetic acid sodium salt
MWCO: molecular-weight cut off value
SDS-PAGE: sodium dodecyl sulfate polyacrylamide gel-electrophoresis
AOX-1: *P. pastoris* alcohol oxidase 1
UV–Vis: ultraviolet–visible
