# Supplementary material for: Preparation of a functional fluorescent human Fas ligand extracellular domain derivative using a three-dimensional structure guided site-specific fluorochrome conjugation
Source: Springerplus. 2016 Jul 7;5(1):997. doi: 10.1186/s40064-016-2673-8 (PMC4936993; doi:10.1186/s40064-016-2673-8)
Supplement: Supplementary file 1 — 10.1186/s40064-016-2673-8 Stereo-view of three-dimensional hFasLECD structure. [file 40064_2016_2673_MOESM1_ESM.pptx]

## Slide 1
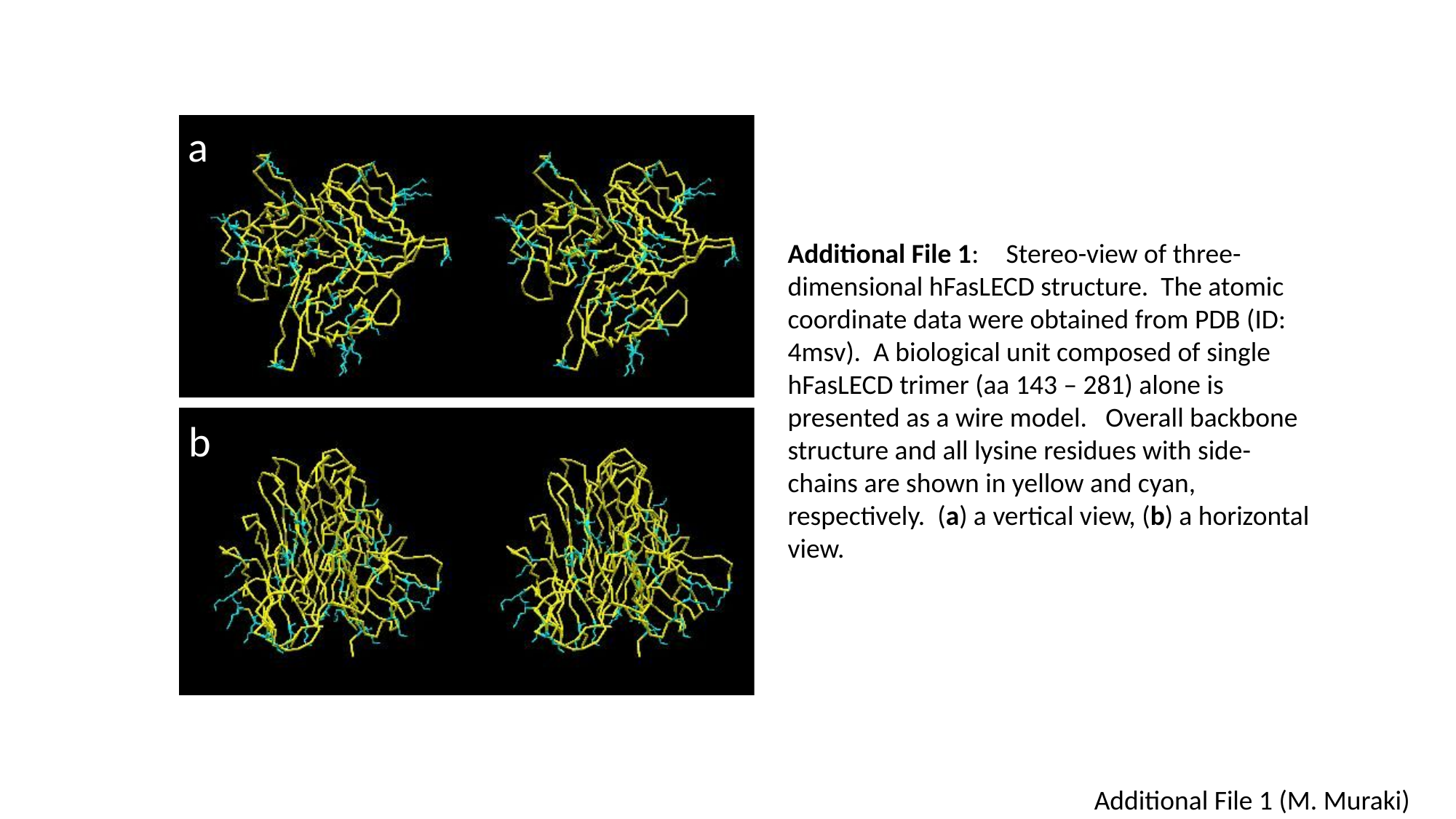

a
b
Additional File 1:	Stereo-view of three-dimensional hFasLECD structure. The atomic coordinate data were obtained from PDB (ID: 4msv). A biological unit composed of single hFasLECD trimer (aa 143 – 281) alone is presented as a wire model. Overall backbone structure and all lysine residues with side-chains are shown in yellow and cyan, respectively. (a) a vertical view, (b) a horizontal view.
Additional File 1 (M. Muraki)
