# Supplementary material for: Preparation of a functional fluorescent human Fas ligand extracellular domain derivative using a three-dimensional structure guided site-specific fluorochrome conjugation
Source: Springerplus. 2016 Jul 7;5(1):997. doi: 10.1186/s40064-016-2673-8 (PMC4936993; doi:10.1186/s40064-016-2673-8)
Supplement: Supplementary file 2 — 10.1186/s40064-016-2673-8 Analysis of molecular-weight size markers by size-exclusion chromatography. [file 40064_2016_2673_MOESM2_ESM.pptx]

## Slide 1
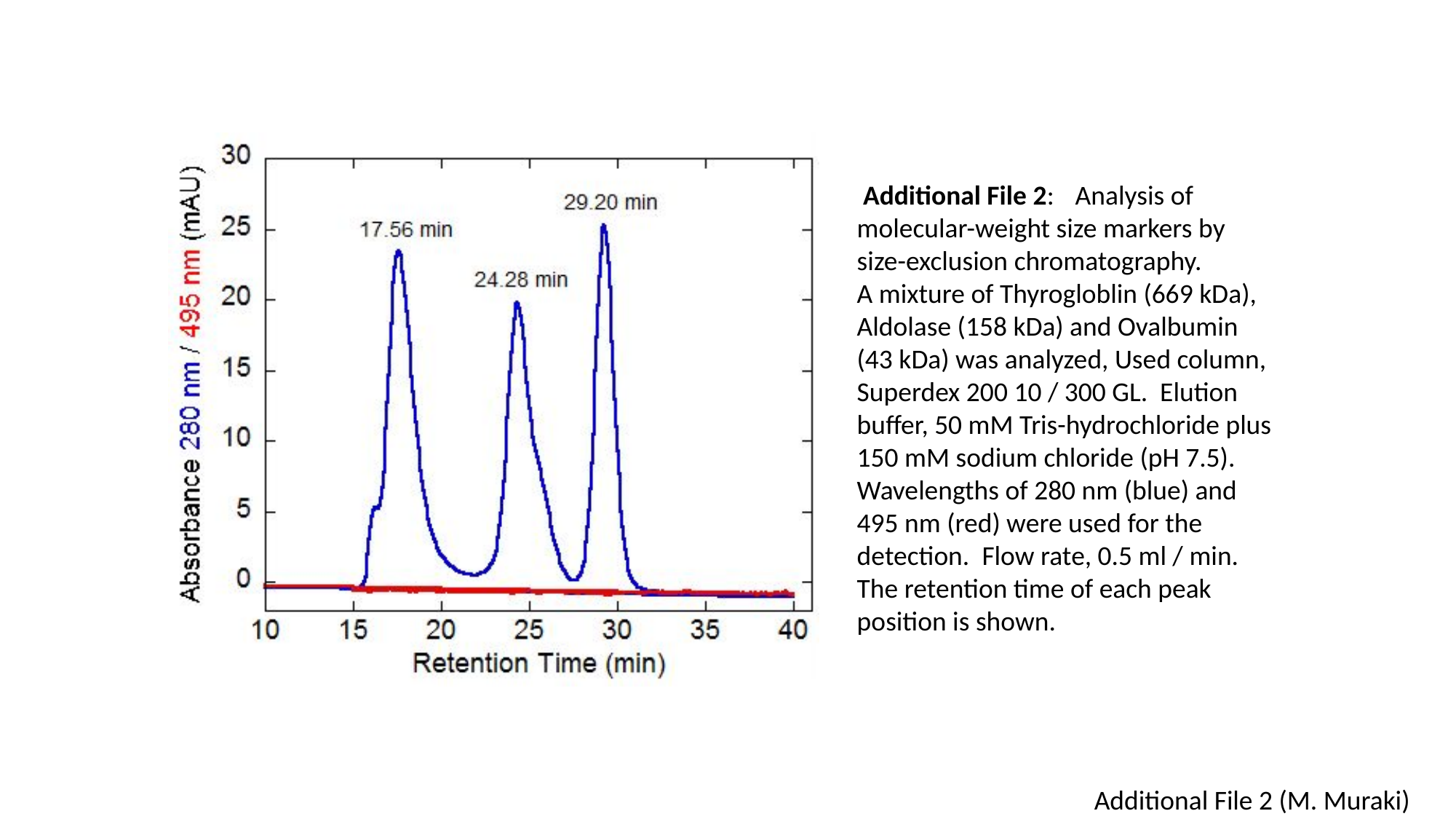

Additional File 2:	Analysis of molecular-weight size markers by size-exclusion chromatography.
A mixture of Thyrogloblin (669 kDa), Aldolase (158 kDa) and Ovalbumin (43 kDa) was analyzed, Used column, Superdex 200 10 / 300 GL. Elution buffer, 50 mM Tris-hydrochloride plus 150 mM sodium chloride (pH 7.5). Wavelengths of 280 nm (blue) and 495 nm (red) were used for the detection. Flow rate, 0.5 ml / min. The retention time of each peak position is shown.
Additional File 2 (M. Muraki)
