# Supplementary material for: Preparation of a functional fluorescent human Fas ligand extracellular domain derivative using a three-dimensional structure guided site-specific fluorochrome conjugation
Source: Springerplus. 2016 Jul 7;5(1):997. doi: 10.1186/s40064-016-2673-8 (PMC4936993; doi:10.1186/s40064-016-2673-8)
Supplement: Supplementary file 3 — 10.1186/s40064-016-2673-8 Cation-exchange chromatography profile of the purified sample. [file 40064_2016_2673_MOESM3_ESM.pptx]

## Slide 1
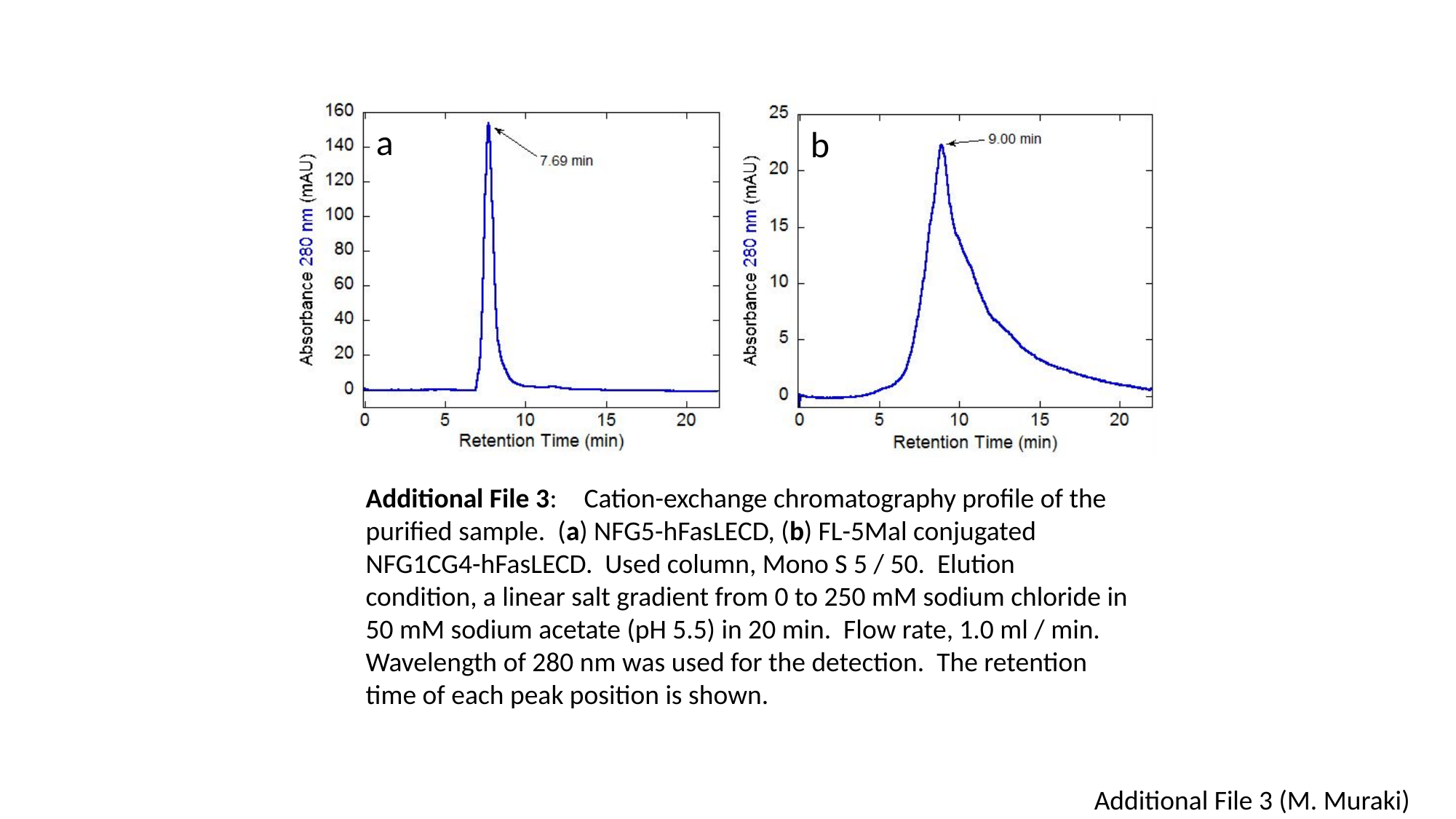

a
b
Additional File 3:	Cation-exchange chromatography profile of the purified sample. (a) NFG5-hFasLECD, (b) FL-5Mal conjugated NFG1CG4-hFasLECD. Used column, Mono S 5 / 50. Elution condition, a linear salt gradient from 0 to 250 mM sodium chloride in 50 mM sodium acetate (pH 5.5) in 20 min. Flow rate, 1.0 ml / min.
Wavelength of 280 nm was used for the detection. The retention time of each peak position is shown.
Additional File 3 (M. Muraki)
